# Associations between fibroblast growth factor 23 and cardiovascular disease in children and adolescents: a systematic review and meta-analysis

**DOI:** 10.3389/fped.2026.1682239

**Published:** 2026-01-27

**Authors:** Jia Na, Zhen Zhen, Wen Yu, Xi Chen, Xia Yu, Yanyan Xiao, Yue Yuan

**Affiliations:** Department of Cardiology, Beijing Children’s Hospital Capital Medical University, National Center for Children’s Health, Beijing, China

**Keywords:** adolescents, cardiovascular diseases, children, fibroblast growth factor 23, meta-analysis, systematic review

## Abstract

**Objective:**

Over the previous decade, fibroblast growth factor 23 (FGF23) has been identified as a key biomarker in the context of cardiovascular diseases(CVD). The primary goal of this investigation was to determine the association between FGF23 and the susceptibility to CVD among children and adolescents.

**Methods:**

We performed an electronic search of the Cochrane Library, PubMed, Web of Science, and Embase databases, covering the period from their inception until August 4, 2022. The random effects model was applied. Additionally, we conducted stratified analyses and performed a sensitivity analysis as part of our further investigation.

**Results:**

A total of 11 studies involving 1,428 participants, including 366 individuals with cardiovascular disease and 1,062 control subjects, were included in the analysis. Children and adolescents with cardiovascular disease exhibited significantly higher serum FGF-23 levels compared to the control group [standardized mean difference [SMD] = 1.28, 95% confidence interval [CI] 0.53–2.03; *I*^2^ = 93.0%], as determined using a random-effects model. In categorical analyses across six studies, the pooled odds ratio did not demonstrate a statistically significant association with disease risk [odds ratio (OR) = 1.64, 95% CI 0.86–3.12; *I*^2^ = 100.0%]. Meta-regression analysis, accounting for variables such as type of cardiovascular disease, assay type, chronic kidney disease (CKD) status, and CKD stage, yielded a restricted maximum likelihood (REML) estimate of (*τ*^2^ = 0.2321) for the SMD outcome, indicating residual heterogeneity (*I*^2^_res ≈ 70.3%) and an adjusted R^2^ of 83.6%. The joint test for covariates was not statistically significant (Knapp–Hartung corrected Prob > F = 0.3165). For the categorical outcome, the meta-regression analysis produced a boundary estimate (*τ*^2^ = 0) with *I*^2^_res = 0% and a non-significant joint test (Prob > F = 0.3479); however, these findings are likely influenced by the limited number of studies and restricted degrees of freedom.

**Conclusion:**

Serum FGF-23 levels are elevated in pediatric populations with cardiovascular disease, but study-specific thresholds have not shown a clear independent association with risk. The variability in findings, reliance on observational study designs, and differences in assay methods contribute to the uncertainty about its prognostic value. Therefore, standardized prospective studies reporting on renal function and mineral metabolism markers are needed.

**Systematic Review Registration:**

https://www.crd.york.ac.uk/PROSPERO/view/CRD42023480899, PROSPERO CRD42023480899.

## Introduction

Cardiovascular disease (CVD) stands as the primary global cause of mortality, accounting for approximately 17.9 million deaths, equivalent to roughly one-third of all global fatalities (1, 2). Across the past several decades, there has been a consistent uptick in the prevalence of obesity and other risk factors associated with CVD in children, observed in both developed and developing countries (3). These increases can be attributed to significant factors such as reduced physical activity levels and the consumption of high-calorie, low-nutrient-density diets (4). Consequently, CVD, which is presently on the rise worldwide, has emerged as a significant public health challenge in the 21st century (5). Among children and adolescents, there is a growing adoption of unhealthy lifestyle practices, and this is a contributing factor to the overall elevation of cardiovascular risk in this population (6). Multiple prospective investigations have highlighted that risk factors for CVD during childhood have a tendency to endure into adulthood (7, 8).

It is widely recognized that fibroblast growth factor 23 (FGF23) plays a crucial role as a regulator of phosphate and vitamin D, and it is indispensable for mineral and bone metabolism (9). The FGF23 protein consists of 251 amino acids, representing the full-length form of a 32-kilodalton (kDa) protein (10). FGF23 belongs to the FGF family and includes the N-terminal segment responsible for processing (11), as well as the C-terminal segment that interacts with *α*-Klotho (12). It is primarily produced and released by osteocytes and osteoblasts. The intact FGF23 (iFGF23) protein is enzymatically cleaved by subtilisin-like proprotein convertases at the Arg179 and Ser180 position, yielding inactive N- and C-terminal fragments (13). Three variations can be detected in the blood: N-terminal FGF23, C-terminal FGF23 (cFGF23), and the intact, full-length iFGF23. It is believed that only the intact iFGF23 has the capacity to bind to the FGFR1c/α-Klotho complex and initiate downstream signaling pathways (14, 15).

In addition to its established functions, recent studies have revealed possible connections between FGF23 and cardiovascular well-being (16, 17). The influence of FGF23 on cardiac myocytes, vascular cells, and the renin-angiotensin-aldosterone system (18, 19), whether direct or indirect, raises the possibility of its role in the pathogenesis of cardiovascular diseases. Considering the pivotal role of FGF23 in mineral metabolism, it is of utmost significance to investigate its connection with CVD in children and adolescents. Thus, we undertook a systematic review and meta-analysis to explore the association between serum FGF-23 levels and clinical indicators, specifically aiming to assess the utility of FGF-23 as a predictor of CVD in the pediatric and adolescent population.

## Methods

### Search strategy and study selection

In designing the search strategy, the study adhered to the guidelines outlined in the “Cochrane Guidelines for Systematic Reviews of Health Promotion and Public Health Interventions. English-language articles published from their inception until August 4, 2022, were searched for in PubMed, Cochrane Library, Web of Science, and Embase. The search incorporated terms and keywords associated with fibroblast Growth Factor-23, cardiovascular disease, cardiac events, pediatric, and children. The detailed search strategy can be found in “Supplementary Appendix 1: Search strategy.”

### Inclusion and exclusion criteria

The included articles met the following criteria: (1) Participants were children or adolescents; (2) FGF-23 was a primary focus of investigation; and (3) at least one clinical cardiovascular disease outcome was evaluated. Cardiovascular outcomes of interest included myocardial infarction(MI), stroke, heart failure(HF), left ventricular hypertrophy(LVH), high blood pressure(HBP), severe cardiac impairment(SCI), left ventricular mass(LVM), and coronary artery calcification(CAC). Studies were excluded if they met the following criteria: (1) Insufficient data availability; (2) Qualification as a case report, review, abstract, letter, or comment; and (3) Involvement in animal research.

### Data extraction and quality evaluation

Two authors independently collected the following details for each relevant study: primary author, country of origin, publication year, sample size, duration of follow-up, FGF23 categories, reported outcomes, method of FGF23 measurement, and odds ratios(ORs) along with their corresponding 95% confidence intervals (95%CI). The Newcastle-Ottawa Scale (NOS) (20) was employed to assess the quality of the studies included, with those scoring higher than 6, suggesting a moderate-to-high risk of bias, being included in the analysis.

### Statistical analysis

All analyses were conducted using Stata 14.0. For continuous outcomes, we extracted means and standard deviations and calculated standardized mean differences (SMD) with 95% confidence intervals. In cases where only medians and interquartile ranges were reported, we converted these to means and standard deviations using established methodologies. For dichotomous outcomes, we extracted reported odds ratios or event counts and pooled odds ratios with 95% confidence intervals (CIs). Heterogeneity was evaluated using Cochran's *Q* test and the *I*^2^ statistic; an *I*^2^ value greater than 50% necessitated the use of a random-effects model, while a fixed-effects model was employed otherwise (21). Pre-specified subgroup analyses included FGF-23 assay type (intact vs. C-terminal), chronic kidney disease (CKD) status (presence vs. absence), CKD stage, and type of cardiovascular outcomes (LVH or other). Where data permitted, we planned to conduct multivariable meta-regression to assess the synergistic effects of covariates utilized in subgroup analyses on effect estimates. Sensitivity analyses were performed by sequentially excluding individual studies. Publication bias was assessed using funnel plots and both Begg's and Egger's tests; where necessary, trim-and-fill correction was applied, and results were reported accordingly.

## Results

### Literature search and study characteristics

**Figure 1:**
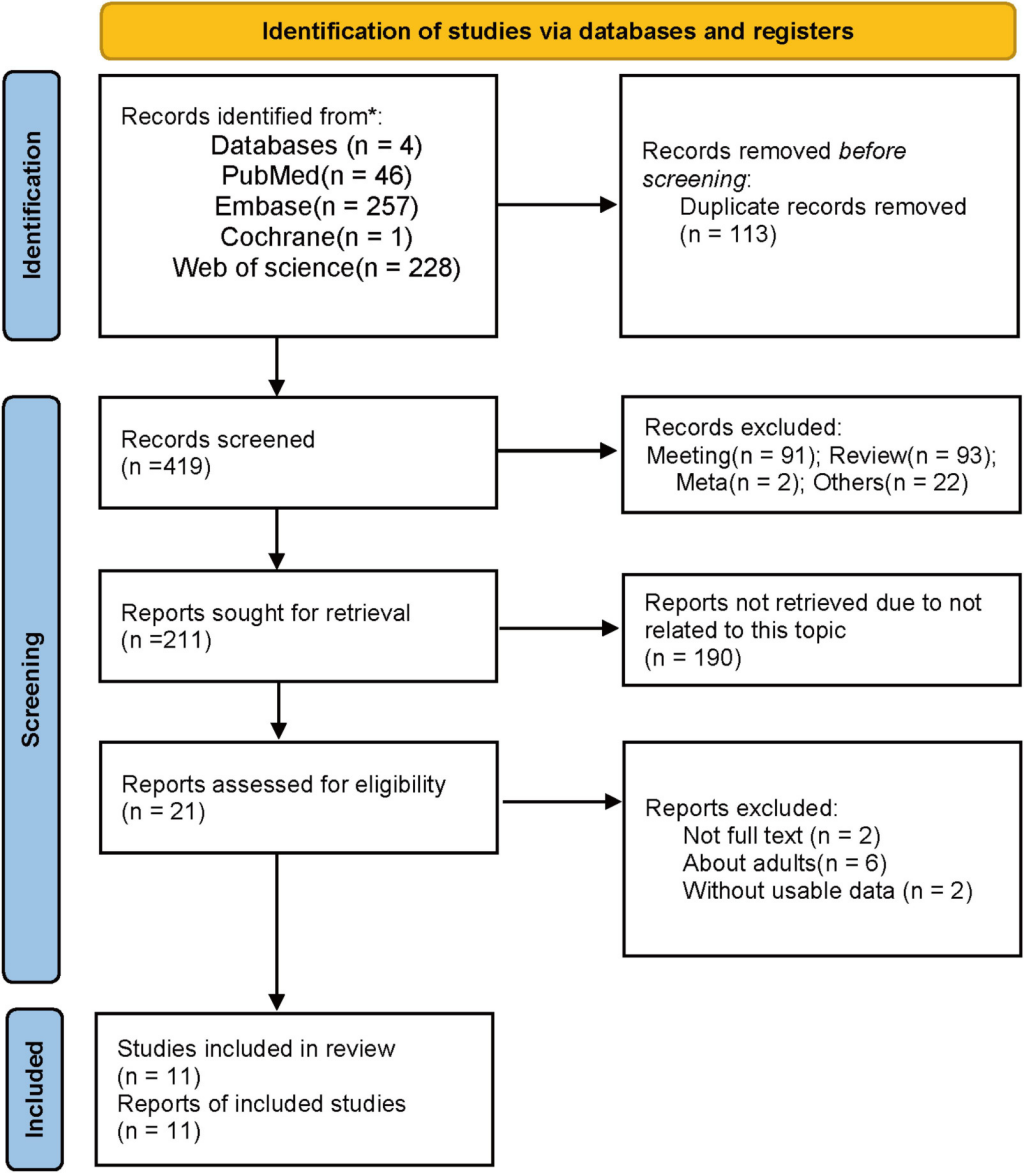
A flow diagram of search results.

Following the initial search conducted across four databases, we identified a total of 532 studies, with 46 originating from PubMed, 257 from Embase, 1 from the Cochrane Library, and 228 from Web of Science (Figure 1). A total of 419 article abstracts were subjected to screening based on the predefined inclusion and exclusion criteria. Twenty-one studies were initially considered eligible and, as a result, underwent a thorough full-text assessment. Eleven studies (22–32) were eligible for inclusion after a careful review of their full texts. The main characteristics of the included studies are outlined in Table 1. Every study that met the inclusion criteria was published during the period from 2013 to 2023. Out of four continents, Asia and North America had the highest number of published studies (*n* = 4), followed by Africa (*n* = 2), and the least was from Europe (*n* = 1). Across the studies, the sample size varied from 30 to 587, with a cumulative total of 1,428 participants. Among the included studies, six examined C-terminal fragments of FGF23, while four measured intact FGF23.

**Table 1:** Characteristics of the studies included in this meta-analysis.

Author	Year	Country	Participants		The type of CAD	FGF-23 sample	FGF23 measurement	CKD status (stage)	Follow up (Y, M)	Outcomes assessed
			Events	Controls						
Isakova (22)	2013	USA	20	17	HF	c-terminal	ELISA	non-CKD	NA	SMD
Ali (23)	2014	USA	14	96	LVH	c-terminal	NA	non-CKD	NA	SMD
Sinha (24)	2015	UK	13	17	LVH	intact	ELISA	CKD (Ⅲ–Ⅴ)	6.5Y	SMD
Falkner (25)	2017	USA	48	140	LVH	c-terminal	ELISA	not reported	NA	SMD
Mudi (26)	2017	South Africa	24	64	LVH	intact	ELISA	CKD (Ⅰ–Ⅴ)	NA	SMD, OR
Mitsnefes (27)	2018	USA	67	520	LVH	c-terminal	ELISA	CKD (Ⅰ–Ⅳ)	6–9M	SMD, OR
Lin (28)	2019	China	35	63	HBP	c-terminal	ELISA	not reported	NA	SMD
Palupi-Baroto (29)	2021	Indonesia	5	38	SCI	c-terminal	ELISA	CKD (Ⅱ–Ⅴ)	NA	OR
Singh (30)	2022	India	42	17	LVM	intact	ELISA	CKD (Ⅱ–Ⅴ)	NA	OR
Elzayat (31)	2023	Egypt	40	20	HF	intact	ELISA	non-CKD	12M	SMD, OR
Zhu (32)	2023	China	58	70	CAC	NA	ELISA	CKD (Ⅰ–Ⅴ)	NA	SMD, OR

HF, heart failure; LVH, left ventricular hypertrophy; HBP, high blood pressure; SCI, severe cardiac impairment; LVM, left ventricular mass; CAC, coronary artery calcification; ELISA, Enzyme linked immunosorbent assay; Y, year; M, month; SMD, Standard Mean Difference; OR, odds ratio; NA, not available.

### Study quality

According to the Newcastle-Ottawa Scale, the 11 observational studies included in this analysis were assessed as moderate to high quality, with 8 studies scoring 7 points and 3 studies scoring 8 points. However, a notable limitation is the lack of reporting or insufficient clarification regarding non-response rates in the two groups, which may compromise the assessment of selection bias (Table 2).

**Table 2:** Methodological quality of observational studies included in the meta-analysis^a^.

First author	Representativeness of the cases	Selection of the controls	Ascertainment of exposure	Outcome of interest not present at start of study	Control for important factor or additional factor	Outcome assessment	Same method of ascertainment for cases and controls	Same non-Response for both groups	Total quality scores
Isakova/2013 (22)	⋆	⋆	⋆	⋆	⋆	⋆	⋆	–	7
Ali/2014 (23)	⋆	⋆	⋆	⋆	⋆	⋆	⋆	–	7
Sinha/2015 (24)	⋆	⋆	⋆	⋆	⋆	⋆	⋆	⋆	8
Falkner/2017 (25)	⋆	⋆	⋆	⋆	⋆	⋆	⋆	–	7
Mudi/2017 (26)	⋆	⋆	⋆	⋆	⋆	⋆	⋆	–	7
Mitsnefes/2018 (27)	⋆	⋆	⋆	⋆	⋆	⋆	⋆	⋆	8
Lin/2019 (28)	⋆	⋆	⋆	⋆	⋆	⋆	⋆	–	7
Palupi-Baroto/2021 (29)	⋆	⋆	⋆	⋆	⋆	⋆	⋆	–	7
Singh/2022 (30)	⋆	⋆	⋆	⋆	⋆	⋆	⋆	–	7
Elzayat/2023 (31)	⋆	⋆	⋆	⋆	⋆	⋆	⋆	⋆	8
Zhu/2023 (32)	⋆	⋆	⋆	⋆	⋆	⋆	⋆	–	7

a A study could be awarded a maximum of one star for each item except for the item Control for important factor or additional factor.

### Comparison of FGF-23 levels between children with and without CVD

**Figure 2:**
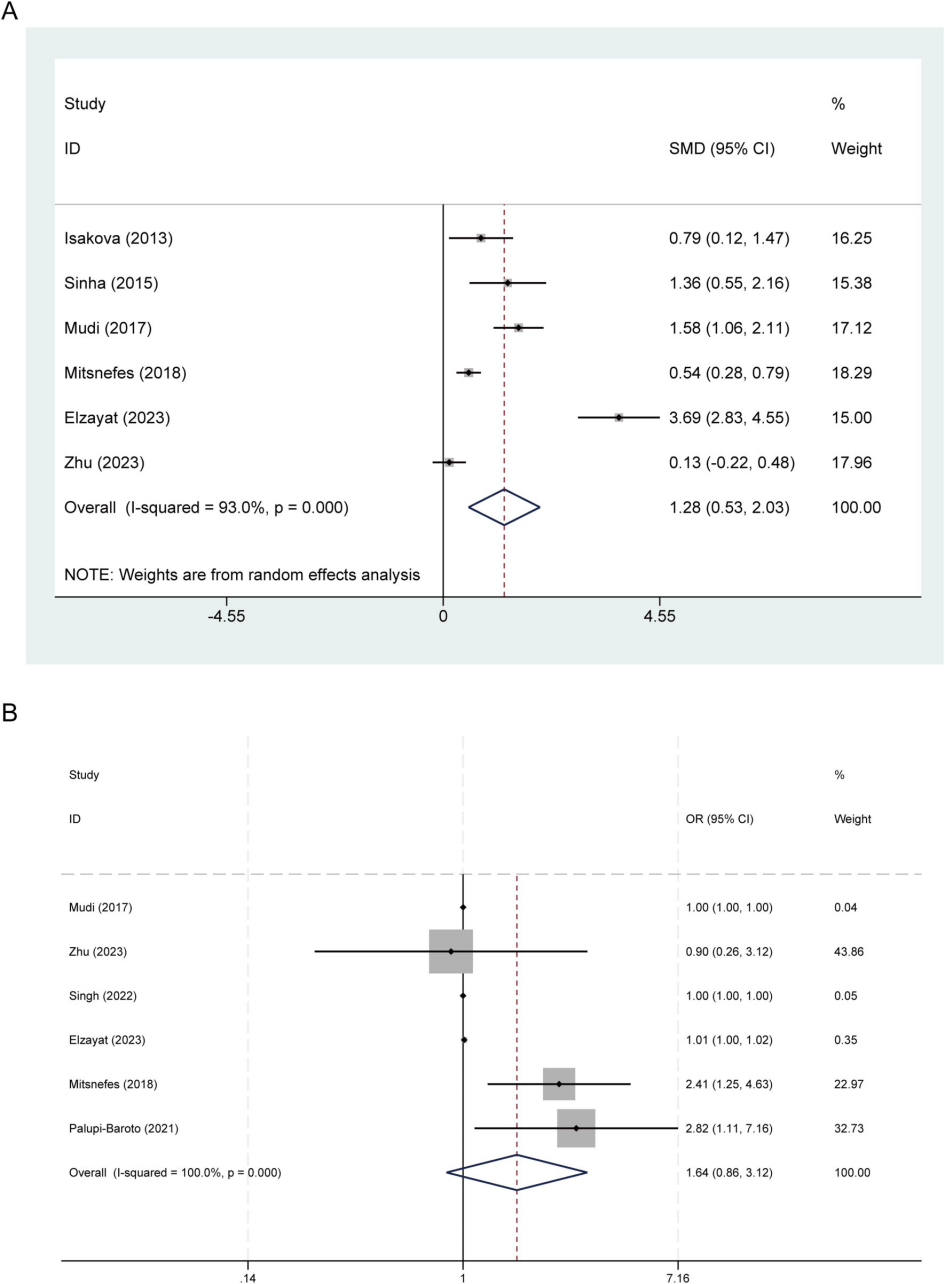
Forest plots of FGF23 and CVDs in children and adolescents. **(A)** FGF-23 levels between children with and without CVD; **(B)** Association between FGF-23 level and risk of CVD.

The pooled random-effects estimate derived from six studies demonstrated that serum FGF-23 levels were significantly elevated in children and adolescents diagnosed with CVD compared to their counterparts without CVD, with a SMD of 1.283 (95% CI: 0.533–2.033), *p* = 0.001. Notably, there was substantial heterogeneity among the studies, as indicated by an *I*^2^ statistic of 93.0% and *p* < 0.001 (Figure 2A).

### Association between FGF-23 level and risk of CVD

In the categorical analysis, six studies comprising 236 cases and 729 controls, assessed the relationship between elevated FGF-23 levels and the risk of CVD. The pooled OR was 1.64 (95% CI: 0.86–3.12, *p* = 0.133), indicating no statistically significant association. Substantial heterogeneity among the studies was observed (*I*^2^ = 100.0%; *p* < 0.001) (Figure 2B).

### Subgroup analysis

**Figure 3:**
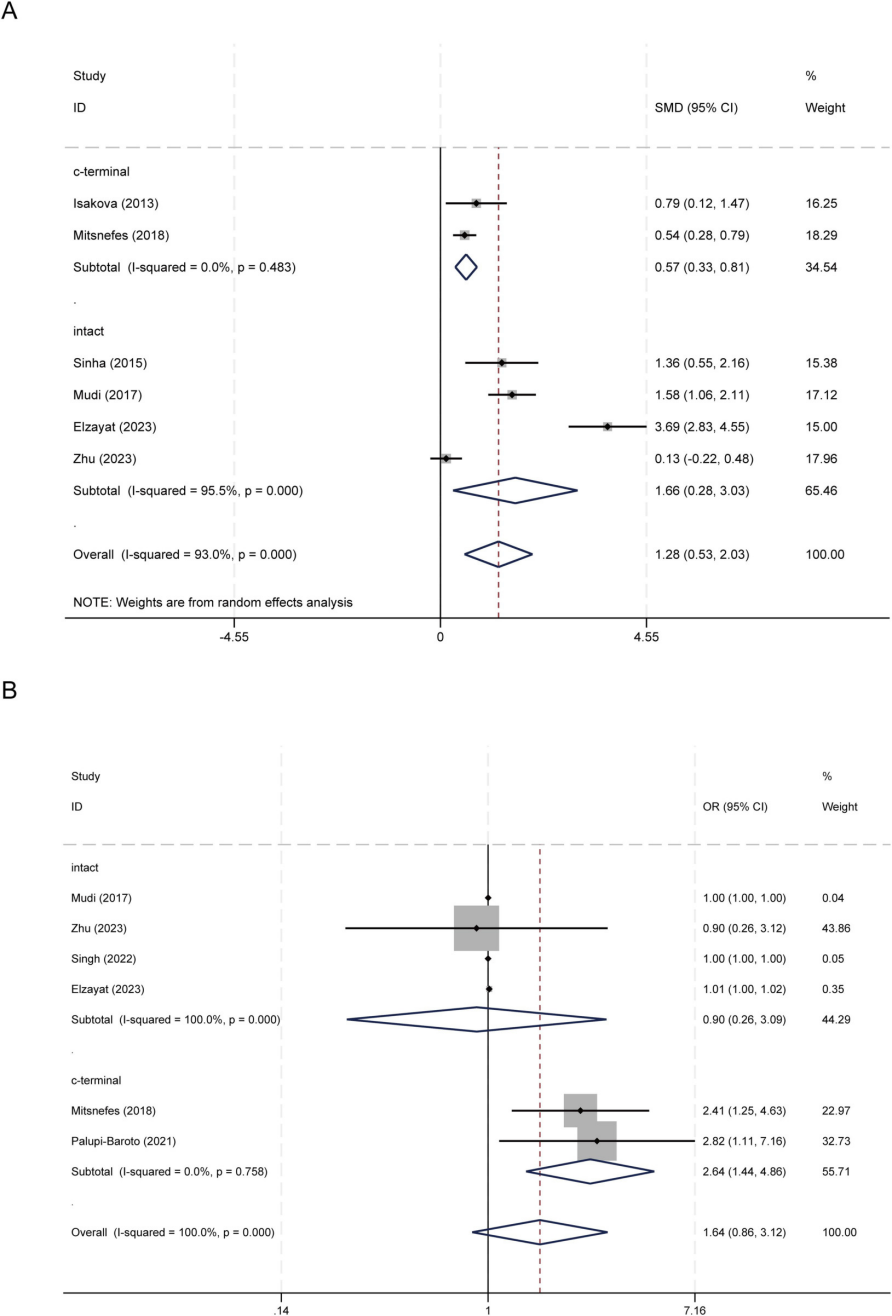
Subgroup analysis based on FGF23 assay type. **(A)** FGF-23 levels between children with and without CVD; **(B)** Association between FGF-23 level and risk of CVD.

Subgroup analyses by FGF-23 assay type, chronic kidney disease (CKD) status, and CKD stage revealed consistent yet heterogeneous patterns. By assay type, both C-terminal and intact FGF-23 levels were higher in CVD cases. C-terminal studies showed a pooled standardized mean difference (SMD) of 0.568 (95% CI: 0.329–0.808; *I*^2^ = 0.0%), while intact studies had a pooled SMD of 1.655 (95% CI: 0.280–3.031; *I*^2^ = 95.5%) (Figure 3A). For categorical associations, the C-terminal subgroup demonstrated a significant pooled effect of OR = 2.643 (95% CI: 1.436–4.865; *I*^2^ = 0.0%), whereas the intact subgroup did not (Figure 3B).

**Figure 4:**
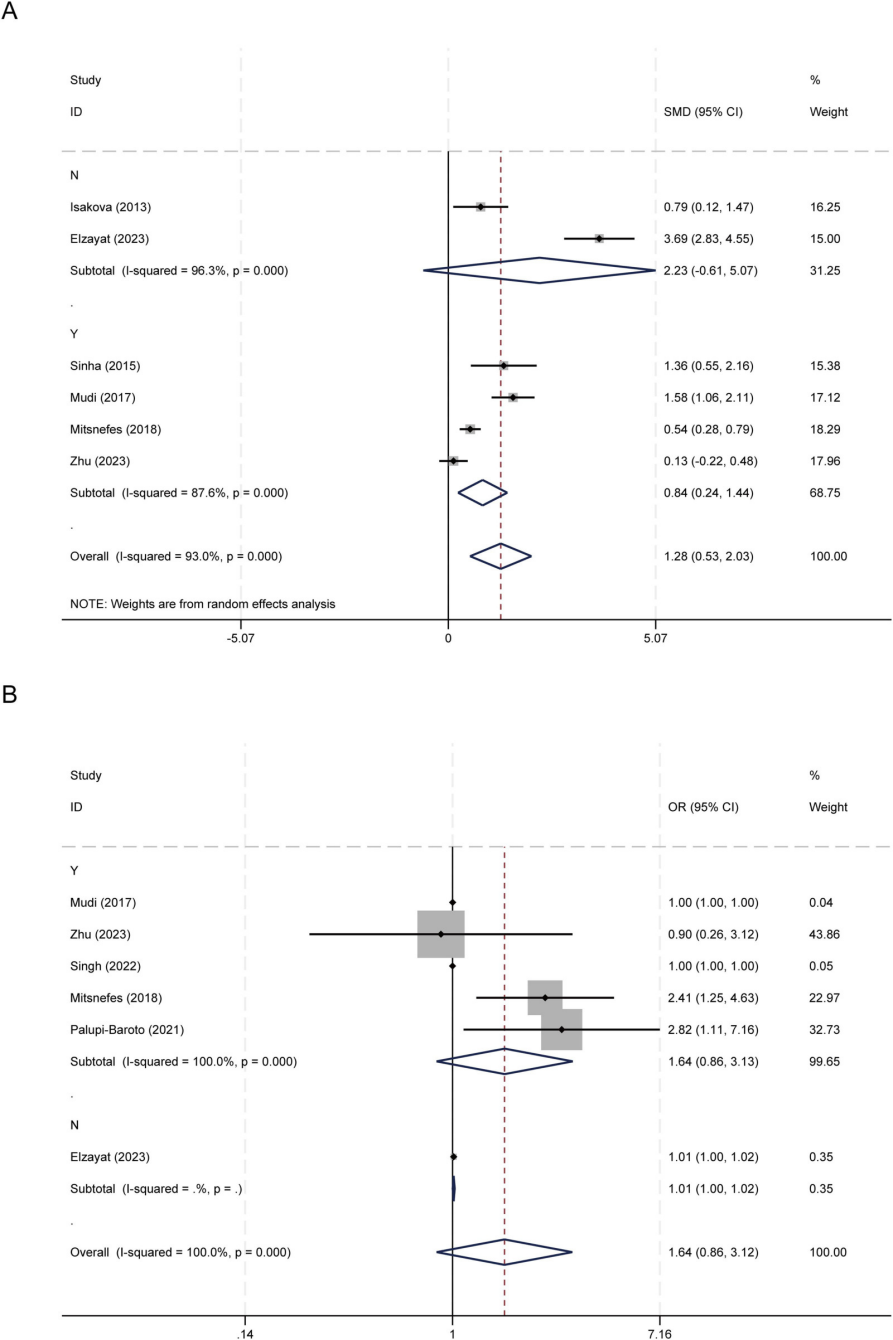
Subgroup analysis based on CKD status. **(A)** FGF-23 levels between children with and without CVD; **(B)** Association between FGF-23 level and risk of CVD.

In terms of CKD status, studies involving patients with CKD yielded a pooled SMD of 0.841 (95% CI: 0.240–1.443; *I*^2^ = 87.6%), while CKD-negative studies produced a larger but imprecise and non-significant SMD of 2.230 (95% CI: −0.611–5.072; *I*^2^ = 96.3%) (Figure 4A). For categorical associations, only one study excluded patients with CKD. The subgroup analysis incorporating patients with CKD produced results consistent with the original combined analysis (OR=1.640; 95% CI: 0.859–3.131; *I*^2^ = 100%); however, the observed heterogeneity was not sufficiently elucidated (Figure 4B).

**Figure 5:**
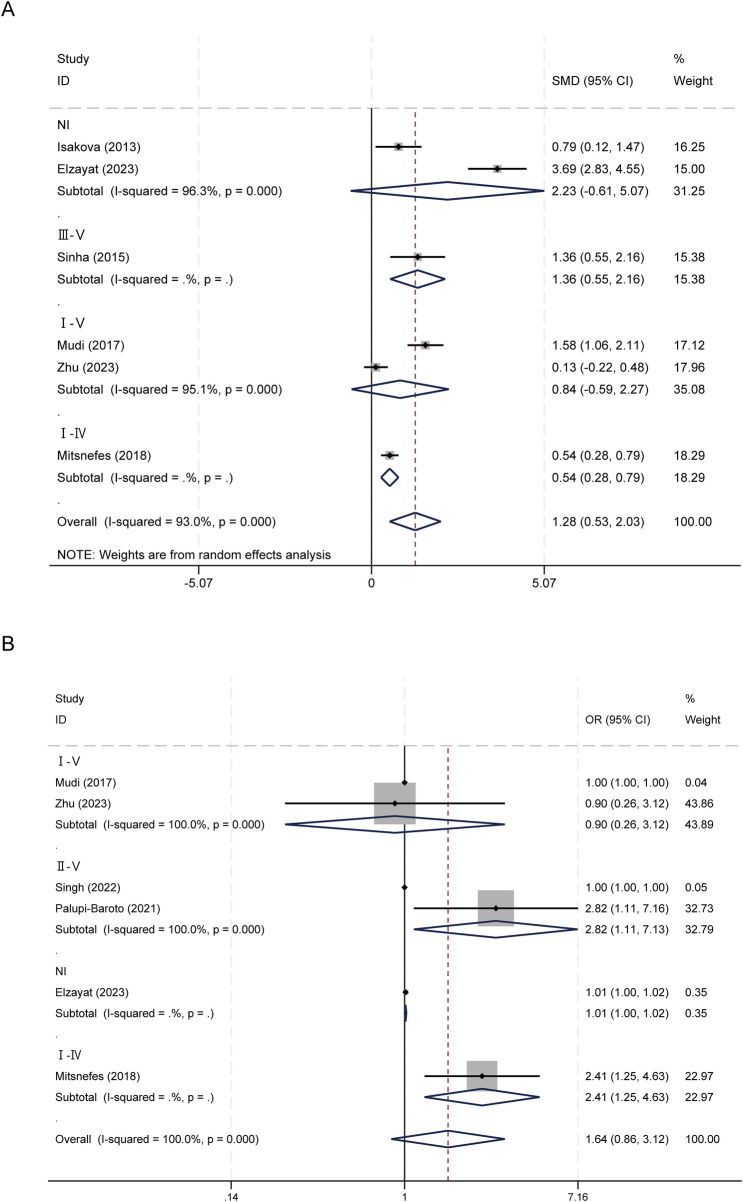
Subgroup analysis based on CKD stage. **(A)** FGF-23 levels between children with and without CVD; **(B)** Association between FGF-23 level and risk of CVD.

Analyses by CKD stage indicated significant elevations in several strata (III–V: SMD 1.356, 95% CI: 0.552–2.161; I–IV: SMD 0.536, 95% CI: 0.279–0.792), though other strata showed inconsistencies (Figure 5A). For categorical associations, based on the extremely high heterogeneity among the studies, the II-V CKD subgroup indicated that FGF-23 increased the risk of CVD (OR=2.815; 95% CI: 1.111–7.134; *I*^2^ = 100%) (Figure 5B).

Overall, assay type and CKD-related factors account for some of the differences observed between studies—C-terminal results were more consistent—yet substantial residual heterogeneity remains in several subgroups, limiting definitive conclusions.

### Sensitivity analysis

**Figure 6:**
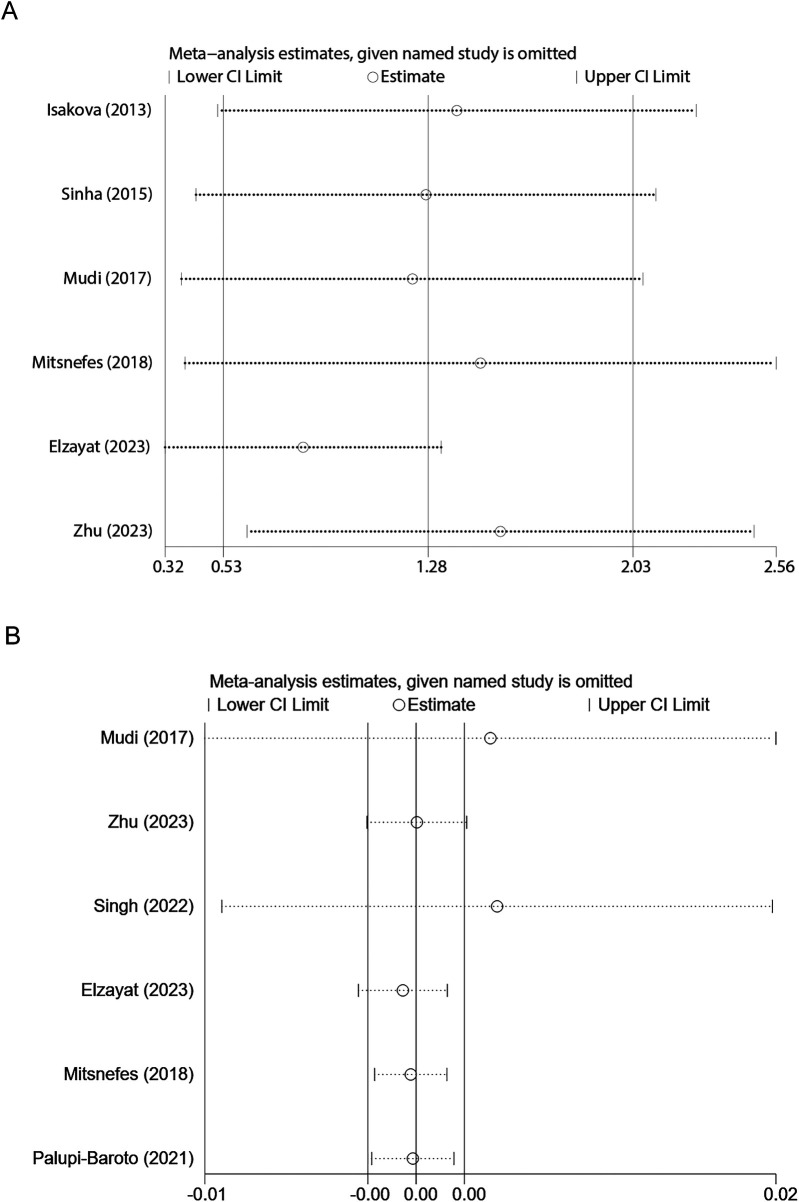
Sensitivity analysis examining the influence of individual studies on pooled results of FGF-23 levels in children and adolescents. **(A)** FGF-23 levels between children with and without CVD; **(B)** Association between FGF-23 level and risk of CVD.

In the sensitivity analysis, the sequential exclusion of individual studies consistently supported the robustness of the overall conclusion. For comparisons involving continuous variables SMD, the combined effect remained within the range of 0.825–1.548 following the removal of any single study, with each associated 95% confidence interval not crossing zero. These findings are consistent with the overall combined SMD of 1.283 (95% CI: 0.533–2.033) (Figure 6A). In the sensitivity analysis of categorical variables, the combined log effect estimate remained approximately 0.00138937 (95% CI: −0.00168673 to 0.00446547) after the exclusion of individual studies, indicating that no single study exerted a significant influence on the overall effect (Figure 6B).

### Publication bias

**Figure 7:**
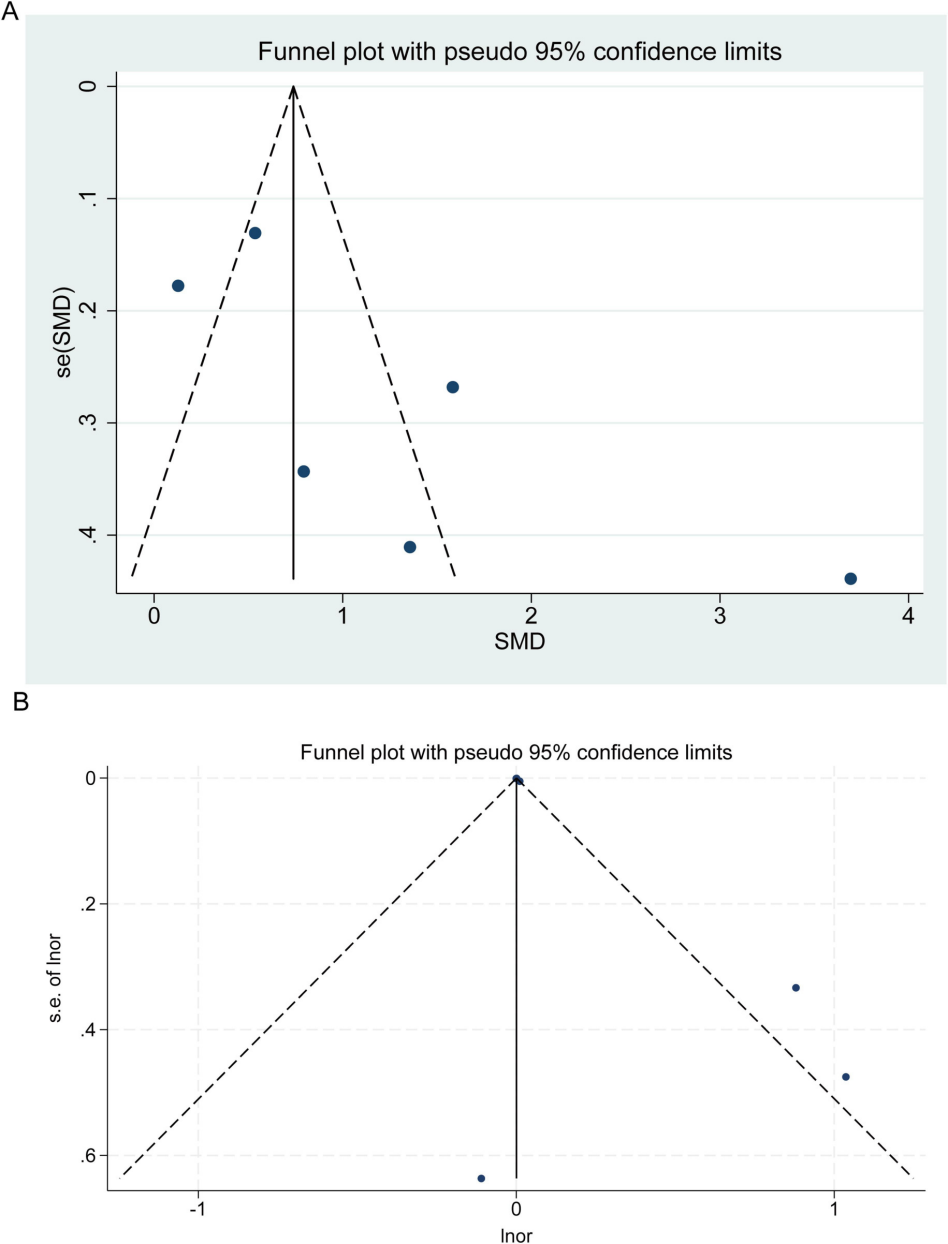
Funnel plot for publication bias of FGF-23 levels in children and adolescents. Each point represents a separate study for the indicated association. **(A)** FGF-23 levels between children with and without CVD; **(B)** Trim and fill analysis.

Funnel plots, along with Begg's and Egger's tests, were conducted to evaluate publication bias within the literature. The funnel plot suggests a potential presence of publication bias (Figure 7); however, the *p*-values obtained from Begg's and Egger's tests do not support the existence of publication bias regarding the continuous (Begg's test *p* = 0.091; Egger's test *p* = 0.093) and categorical outcomes (Begg's test *p* = 0.573; Egger's test *p* = 0.082).

### Meta-regression analyses

In the meta-regression analysis, both models assessed the joint effects of Cvd type, FGF-23 assay, CKD status, and CKD stage on between-study heterogeneity (Supplementary Materials). For the SMD outcome, which included six studies, the Restricted Maximum Likelihood (REML) estimate yielded (tau^2^ = 0.2321), with residual heterogeneity reported as *I*-squared_res = 70.33% and Adj *R*^2^ = 83.60%. The joint test for covariates was not statistically significant following the Knapp-Hartung correction (Prob > *F* = 0.3165), and individual covariate coefficients, such as for FGF-23 assay (2.423), exhibited positive trends but lacked statistical significance. In the case of the odds ratio/natural logarithm of the odds ratio (OR/lnOR) outcome, also comprising six studies, the estimate for (tau^2^) was reported as 0, with *I*-squared_res = 0% and Adj *R*^2^ = 100%. The joint test in this scenario was similarly non-significant (Prob > *F* = 0.3479); however, this boundary estimate is likely affected by the limited number of studies and the consequent reduction in degrees of freedom.

## Discussion

This systematic review and meta-analysis of 11 observational studies with 1,428 participants (366 with cardiovascular disease and 1,062 controls) found that circulating concentrations of FGF-23 were significantly elevated in children and adolescents with cardiovascular disease. However, categorical analyses using study-specific thresholds did not show a statistically significant association with disease risk. Subgroup analyses indicated that the type of assay (C-terminal vs. intact) and CKD status partially explained the between-study differences, although considerable heterogeneity remained.

According to our analysis, children and adolescents with CVD exhibit elevated serum FGF23 levels. Our finding is in harmony with several investigations conducted in children and adults, all of which have established a compelling link between increased FGF23 levels and unfavorable cardiovascular outcomes. In a study by Isakova et al (22), FGF23 levels were compared between 20 children diagnosed with chronic heart failure (HF) resulting from different causes and a control group of 17 healthy individuals. They observed a twofold rise in FGF23 levels in patients in contrast to healthy controls. Additionally, they detected a noteworthy relationship between FGF23 levels and both the clinical severity of HF and left ventricular (LV) dilatation. Notably, these associations were not influenced by estimated glomerular filtration rate (eGFR) (22). Studies conducted in adults demonstrated that FGF23 levels were heightened in patients with HF, whether they had preserved EF (33) or reduced EF (34). In addition, in their research, Andersen et al (35) found that patients with acute decompensated HF had elevated serum levels of FGF23.

On the whole, FGF23 serves as a central player in the intricate web of interactions between the skeletal system and other organs. In the present day, it is widely recognized that bones function as an endocrine organ, as they produce hormones that enable communication with other organs (36). Produced and secreted by various tissues, FGF23 functions as a hormone, with the main contributors being osteocytes and mature osteoblasts (37). Nevertheless, recent research has unveiled that an excess of FGF23 can result in adverse effects on multiple organs, including the heart, bone structure, and endothelium (38–40). The acknowledgment of FGF23's presence and its activities in cardiovascular tissues has ignited interest in its possible ramifications for cardiovascular health. It is proposed that FGF23 might exert both direct and indirect influences on cardiac myocytes and vascular cells (19). Furthermore, FGF23 might have an impact on the renin-angiotensin-aldosterone system (RAAS), which plays a pivotal role in regulating blood pressure and cardiovascular physiology (18). These findings indicate that FGF23 may be involved in the pathogenesis of cardiovascular diseases, including those that affect the pediatric and adolescent population. Contrary to our findings, some investigations in adult populations have shown a substantial independent link between heightened FGF23 levels and the risk of CVD (41, 42). The physiology of FGF-23 in children exhibits several distinctions from that in adults, which may alter cardiovascular associations. Factors such as active bone growth, hormonal changes related to puberty, and the age-dependent expression of FGFRs and *α*-Klotho can significantly influence circulating levels of FGF-23 and tissue responsiveness (43). These developmental considerations, in conjunction with variations in phosphate handling and vitamin D metabolism, suggest that findings derived from adult populations may not be directly applicable to pediatric cohorts (44). Consequently, age or pubertal stage may serve as effect modifiers and should be systematically reported and analyzed in future research endeavors.

Several limitations restrict the certainty and generalizability of our conclusions. First, the studies included in this review are primarily observational and predominantly cross-sectional or short-term, which precludes causal inference. Second, most studies assessed FGF-23 at a single baseline time point without longitudinal evaluation of biomarker trajectories. Third, assay heterogeneity and inconsistent threshold definitions hinder comparability across studies. Fourth, incomplete reporting of renal function and mineral metabolism markers obstructs comprehensive adjustment for key confounders. Fifth, the considerable heterogeneity between studies diminishes confidence in pooled estimates. Finally, although formal tests (Begg and Egger) did not universally indicate publication bias, funnel plots suggested potential small-study effects that cannot be disregarded.

To address these limitations, future research should prioritize the following: prospective cohort designs with adequate follow-up for definitive cardiovascular endpoints; standardized, well-validated FGF-23 assays with clear distinctions between intact vs. C-terminal methods and units; routine measurement and reporting of renal function and mineral metabolism markers; pre-specified stratification by age and pubertal stage; and harmonized threshold definitions where categorical analyses are intended. Where feasible, an individual participant data meta-analysis would facilitate consistent adjustment for confounders and enable exploration of effect modification.

In conclusion, pediatric patients diagnosed with cardiovascular disease exhibit elevated levels of circulating FGF-23 upon continuous measurement; however, categorical analyses employing heterogeneous, study-specific thresholds fail to establish a definitive independent association with disease risk. The current body of evidence is constrained by significant heterogeneity, variability in assay techniques, and the predominance of cohorts enriched with CKD. As a result, the prognostic significance of FGF-23 in children and adolescents remains indeterminate. Therefore, well-structured prospective studies utilizing standardized assays and comprehensive reporting of renal and mineral metabolism parameters are essential to ascertain whether FGF-23 can be regarded as a reliable biomarker for cardiovascular health in the pediatric population.

## Data Availability

The original contributions presented in the study are included in the article/Supplementary Material, further inquiries can be directed to the corresponding authors.
