# Supplementary material for: Associations between fibroblast growth factor 23 and cardiovascular disease in children and adolescents: a systematic review and meta-analysis
Source: Front Pediatr. 2026 Jan 27;14:1682239. doi: 10.3389/fped.2026.1682239 (PMC12886502; doi:10.3389/fped.2026.1682239)
Supplement: Supplementary file 1 [file Table1.docx]

**Search strategy of PubMed**

| No. | Query | Results |
| --- | --- | --- |
| 15 | ((((("Fibroblast Growth Factor-23"[Mesh]) OR (Fibroblast Growth Factor 23[Title/Abstract])) OR (FGF23 protein[Title/Abstract])) AND ((((((((("Cardiovascular Diseases"[Mesh]) OR ((((((((((Cardiovascular Disease[Title/Abstract]) OR (Disease, Cardiovascular[Title/Abstract])) OR (Major Adverse Cardiac Events[Title/Abstract])) OR (Cardiac Events[Title/Abstract])) OR (Cardiac Event[Title/Abstract])) OR (Event, Cardiac[Title/Abstract])) OR (Adverse Cardiac Event[Title/Abstract])) OR (Adverse Cardiac Events[Title/Abstract])) OR (Cardiac Event, Adverse[Title/Abstract])) OR (Cardiac Events, Adverse[Title/Abstract]))) OR (myocardial infarction[Title/Abstract])) OR (stroke[Title/Abstract])) OR (heart failure[Title/Abstract])) OR (atrial fibrillation[Title/Abstract])) OR (coronary heart disease[Title/Abstract])) OR (left ventricular hypertrophy[Title/Abstract])) OR (hypertension[Title/Abstract]))) AND (((((((("Pediatrics"[Mesh]) OR (Pediatric[Title/Abstract])) OR (paediatrics[Title/Abstract])) OR (paediatric[Title/Abstract])) OR (Pediatrics[Title/Abstract])) OR (child[MeSH Terms])) OR (child[Title/Abstract])) OR (children[Title/Abstract]))) NOT (review[Publication Type]) | 46 |
| 14 | review[Publication Type] | 3,200,597 |
| 13 | (((("Fibroblast Growth Factor-23"[Mesh]) OR (Fibroblast Growth Factor 23[Title/Abstract])) OR (FGF23 protein[Title/Abstract])) AND ((((((((("Cardiovascular Diseases"[Mesh]) OR ((((((((((Cardiovascular Disease[Title/Abstract]) OR (Disease, Cardiovascular[Title/Abstract])) OR (Major Adverse Cardiac Events[Title/Abstract])) OR (Cardiac Events[Title/Abstract])) OR (Cardiac Event[Title/Abstract])) OR (Event, Cardiac[Title/Abstract])) OR (Adverse Cardiac Event[Title/Abstract])) OR (Adverse Cardiac Events[Title/Abstract])) OR (Cardiac Event, Adverse[Title/Abstract])) OR (Cardiac Events, Adverse[Title/Abstract]))) OR (myocardial infarction[Title/Abstract])) OR (stroke[Title/Abstract])) OR (heart failure[Title/Abstract])) OR (atrial fibrillation[Title/Abstract])) OR (coronary heart disease[Title/Abstract])) OR (left ventricular hypertrophy[Title/Abstract])) OR (hypertension[Title/Abstract]))) AND (((((((("Pediatrics"[Mesh]) OR (Pediatric[Title/Abstract])) OR (paediatrics[Title/Abstract])) OR (paediatric[Title/Abstract])) OR (Pediatrics[Title/Abstract])) OR (child[MeSH Terms])) OR (child[Title/Abstract])) OR (children[Title/Abstract])) | 65 |
| 12 | ((("Pediatrics"[Mesh]) OR ((Pediatric[Title/Abstract]) OR (paediatrics[Title/Abstract]) OR (paediatric[Title/Abstract]) OR (Pediatrics[Title/Abstract]))) OR (child[MeSH Terms])) OR ((child[Title/Abstract]) OR (children[Title/Abstract])) | 2,728,012 |
| 11 | ("Cardiovascular Diseases"[Mesh]) OR (((((((((((Cardiovascular Disease[Title/Abstract]) OR (Disease, Cardiovascular[Title/Abstract])) OR (Major Adverse Cardiac Events[Title/Abstract])) OR (Cardiac Events[Title/Abstract])) OR (Cardiac Event[Title/Abstract])) OR (Event, Cardiac[Title/Abstract])) OR (Adverse Cardiac Event[Title/Abstract])) OR (Adverse Cardiac Events[Title/Abstract])) OR (Cardiac Event, Adverse[Title/Abstract])) OR (Cardiac Events, Adverse[Title/Abstract]))) OR (myocardial infarction[Title/Abstract])) OR (stroke[Title/Abstract])) OR (heart failure[Title/Abstract])) OR (atrial fibrillation[Title/Abstract])) OR (coronary heart disease[Title/Abstract])) OR (left ventricular hypertrophy[Title/Abstract])) OR (hypertension[Title/Abstract])))) | 3,119,102 |
| 10 | (("Fibroblast Growth Factor-23"[Mesh]) OR ((Fibroblast Growth Factor 23[Title/Abstract]) OR (FGF23 protein[Title/Abstract]))) OR (("Fibroblast Growth Factor-23"[Mesh]) OR ((Fibroblast Growth Factor 23[Title/Abstract]) OR (FGF23 protein[Title/Abstract]))) | 5,074 |
| 9 | (child[Title/Abstract]) OR (children[Title/Abstract]) | 1,511,565 |
| 8 | child[MeSH Terms] | 2,156,456 |
| 7 | (Pediatric[Title/Abstract]) OR (paediatrics[Title/Abstract]) OR (paediatric[Title/Abstract]) OR (Pediatrics[Title/Abstract]) | 456,044 |
| 6 | "Pediatrics"[Mesh] | 63,075 |
| 5 | ((((((((((Cardiovascular Disease[Title/Abstract]) OR (Disease, Cardiovascular[Title/Abstract])) OR (Major Adverse Cardiac Events[Title/Abstract])) OR (Cardiac Events[Title/Abstract])) OR (Cardiac Event[Title/Abstract])) OR (Event, Cardiac[Title/Abstract])) OR (Adverse Cardiac Event[Title/Abstract])) OR (Adverse Cardiac Events[Title/Abstract])) OR (Cardiac Event, Adverse[Title/Abstract])) OR (Cardiac Events, Adverse[Title/Abstract]))) OR (myocardial infarction[Title/Abstract])) OR (stroke[Title/Abstract])) OR (heart failure[Title/Abstract])) OR (atrial fibrillation[Title/Abstract])) OR (coronary heart disease[Title/Abstract])) OR (left ventricular hypertrophy[Title/Abstract])) OR (hypertension[Title/Abstract]))) | 1,286,120 |
| 4 | "Cardiovascular Diseases"[Mesh] | 2,723,701 |
| 3 | ("Fibroblast Growth Factor-23"[Mesh]) OR ((Fibroblast Growth Factor 23[Title/Abstract]) OR (FGF23 protein[Title/Abstract])) | 5,074 |
| 2 | (Fibroblast Growth Factor 23[Title/Abstract]) OR (FGF23 protein[Title/Abstract]) | 3,695 |
| 1 | "Fibroblast Growth Factor-23"[Mesh] | 3,766 |

**Search strategy of EMBASE**

| No. | Query | Results |
| --- | --- | --- |
| #11. | #8 AND #9 AND #10 | 257 |
| #10. | #5 OR #6 OR #7 | 4,104,373 |
| #9. | #3OR #4 | 5,774,476 |
| #8. | #1 OR #2 | 9,781 |
| #7. | 'child'/exp | 3,485,494 |
| #6. | pediatrics:ti,ab,kw OR pediatric:ti,ab,kw OR child:ti,ab,kw OR children:ti,ab,kw | 2,350,329 |
| #5. | 'pediatrics'/exp | 142,809 |
| #4. | 'cardiovascular disease'/exp | 5,597,225 |
| #3. | 'cardiovascular diseases':ti,ab,kw OR 'myocardial infarction':ti,ab,kw OR stroke:ti,ab,kw OR 'heart failure':ti,ab,kw OR 'atrial fibrillation':ti,ab,kw OR 'coronary heart disease':ti,ab,kw OR 'left ventricular hypertrophy':ti,ab,kw OR hypertension:ti,ab,kw | 1,934,189 |
| #2. | 'fibroblast growth factor 23'/exp | 9,295 |
| #1. | 'fibroblast growth factor-23':ti,ab,kw OR 'fibroblast growth factor 23':ti,ab,kw OR 'fgf23 protein':ti,ab,kw | 5,368 |

**Search strategy of Cochrane Controlled Register of Trials (CENTAL)**

| NO. | Search deatiles | Hits |
| --- | --- | --- |
| #1 | MeSH descriptor: [Fibroblast Growth Factor-23] explode all trees MeSH | 207 |
| #2 | ("fibroblast growth factor 23"):ti,ab,kw OR (FGF23 protein):ti,ab,kw | 567 |
| #3 | MeSH descriptor: [Cardiovascular Diseases] explode all trees MeSH | 151385 |
| #4 | (Cardiovascular Diseases):ti,ab,kw | 22894 |
| #5 | (Major Adverse Cardiac Events):ti,ab,kw | 7299 |
| #6 | (myocardial infarction):ti,ab,kw | 35652 |
| #7 | (stroke):ti,ab,kw | 68559 |
| #8 | (heart failure):ti,ab,kw | 44067 |
| #9 | (atrial fibrillation):ti,ab,kw | 15695 |
| #10 | (coronary heart disease):ti,ab,kw | 24145 |
| #11 | (left ventricular hypertrophy):ti,ab,kw | 2412 |
| #12 | (hypertension):ti,ab,kw | 71474 |
| #13 | MeSH descriptor: [Pediatrics] explode all trees MeSH | 1179 |
| #14 | MeSH descriptor: [Child] explode all trees MeSH | 78301 |
| #15 | (Pediatric):ti,ab,kw | 40052 |
| #16 | (paediatric):ti,ab,kw | 40044 |
| #17 | (child):ti,ab,kw | 178443 |
| #18 | (children):ti,ab,kw | 178441 |
| #19 | #1 or #2 | 567 |
| #20 | #3 or #4 or #5 or #6 or #7 or #8 or #9 or #10 or #11 or #12 | 271783 |
| #21 | #13 or #14 or #15 or #16 or #17 or #18 | 186945 |
| #22 | #19 and #20 and #21 | 1 |

**Search strategy of Web of science**

**1:TS=("Fibroblast Growth Factor-23" OR "Fibroblast Growth Factor 23" OR "FGF23 protein") NOT DATABASE:("Preprint Citation Index")**

**Run date: Thu Aug 17 2023 15:24:44 GMT+0800; Results: 11,616**

**2: TS=("Cardiovascular Diseases" OR "Major Adverse Cardiac Events" OR "myocardial infarction" OR "stroke" OR "heart failure" OR "atrial fibrillation" OR "coronary heart disease" OR "left ventricular hypertrophy" OR "hypertension") NOT DATABASE:("Preprint Citation Index")**

**Run date: Thu Aug 17 2023 15:26:29 GMT+0800; Results: 4,213,020**

**3: TS=("Pediatrics" OR "Pediatric" OR "child" OR "children") NOT DATABASE:("Preprint Citation Index")**

**Run date: Thu Aug 17 2023 15:27:54 GMT+0800; Results: 4,988,213**

**4: #1 AND #2 AND #3 NOT DATABASE:("Preprint Citation Index")**

**Run date: Thu Aug 17 2023 15:29:14 GMT+0800; Results: 228**

**Meta-regression analysis comparing the FGF-23 levels between children with and without CVD：**

Meta-regression Number of obs = 6

REML estimate of between-study variance tau2 = .2321

% residual variation due to heterogeneity I-squared_res = 70.33%

Proportion of between-study variance explained Adj R-squared = 83.60%

Joint test for all covariates Model F(4,1) = 5.19

With Knapp-Hartung modification Prob > F = 0.3165

---------------------------------------------------------------------------

_ES | Coefficient Std. err. t P>|t| [95% conf. interval]

-------------+-------------------------------------------------------------

Cvdtype | 1.830161 .6540119 2.80 0.218 -6.479848 10.14017

FGF23assay | 2.423008 .7398618 3.27 0.189 -6.977828 11.82384

Ckdstatus | 4.999605 1.595816 3.13 0.197 -15.27716 25.27637

ckdstage | .8476838 .565145 1.50 0.374 -6.333165 8.028532

_cons | -14.09148 4.841062 -2.91 0.211 -75.60301 47.42004

---------------------------------------------------------------------------

**Meta-Regression Analysis of the Association Between FGF-23 Levels and Cardiovascular Disease Risk**

Meta-regression Number of obs = 6

REML estimate of between-study variance tau2 = 0

% residual variation due to heterogeneity I-squared_res = 0.00%

Proportion of between-study variance explained Adj R-squared = 100.00%

Joint test for all covariates Model F(4,1) = 4.23

With Knapp-Hartung modification Prob > F = 0.3479

---------------------------------------------------------------------------

lnor | Coefficient Std. err. t P>|t| [95% conf. interval]

-------------+-------------------------------------------------------------

Cadtype | .0357579 .4289845 0.08 0.947 -5.415007 5.486522

FGF23assay | -.9193362 .3074926 -2.99 0.205 -4.8264 2.987728

Ckdstatus | -.0268166 .4290162 -0.06 0.960 -5.477984 5.424351

ckdstage | -.0004996 .0004596 -1.09 0.473 -.0063393 .00534

_cons | 1.832229 .6150149 2.98 0.206 -5.982276 9.646735

---------------------------------------------------------------------------
